# Water Distribution in the Body as a Source of Information on Markers of Skeletal Muscle Ageing

**DOI:** 10.3390/nu18111818

**Published:** 2026-06-04

**Authors:** Anna Skrzek, Małgorzata Słowinska-Lisowska, Agnieszka Dębiec-Bąk, Agnieszka Matylda Schlichtinger

**Affiliations:** 1 Faculty of Physiotherapy, Department of Physiotherapy in Movment System Dysfunctions and Kinesiology, University of Health and Sports Science in Wrocław, 51-612 Wrocław, Poland; anna.skrzek@awf.wroc.pl; 2 Faculty of Physical Education and Sport, Department of Biological Pronciples of Physical Activity, University of Health and Sports Science in Wrocław, 51-612 Wrocław, Poland; malgorzata.slowinska-lisowska@awf.wroc.pl; 3 Faculty of Finance and Management, WSB Merito University in Wrocław, 53-609 Wrocław, Poland; agnieszka.schlichtinger@wroclaw.merito.pl

**Keywords:** sarcopenia, skeletal muscle ageing, body water distribution

## Abstract

Background/Objectives: Body water, including its distribution between the intracellular (ICW) and extracellular (ECW) compartments, plays an important role in skeletal muscle physiology. The aim of this study was to assess whether body water distribution indices, namely ECW/ICW and ECW/TBW, differ according to age and sex, and whether they are associated with skeletal muscle index (SMI) after adjustment for BMI, age, and sex. Methods: The study included 188 healthy individuals: 71 older women (71.6 ± 6.4 years), 37 older men (72.7 ± 4.7 years), 40 female students (22.5 ± 0.9 years), and 40 male students (22.8 ± 1.8 years). Body composition was assessed using a Tanita MC-780 bioimpedance analyser. The ECW/ICW and ECW/TBW ratios were calculated. Nonparametric analyses, Kendall’s rank correlations, and linear regression models adjusted for BMI, age, and sex were used. Results: Older adults had significantly higher ECW/ICW and ECW/TBW values than younger participants, and women had higher values than men within the same age groups. Both indices correlated positively with age and BMI, and negatively with SMI. In multivariate models, ECW/TBW remained negatively associated with SMI after adjustment. BMI and male sex correlated positively with SMI, whereas age was not a significant predictor. Conclusions: Body water distribution indices (ECW/ICW, ECW/TBW) reflect age- and sex-related differences in water distribution and correlate with SMI independently of BMI, age, and sex. They may serve as complementary markers of skeletal muscle ageing.

## 1. Introduction

In ageing research, alongside skeletal muscle mass, increasing attention is being paid to muscle quality, which in bioelectrical impedance analysis (BIA) can be partly reflected by the distribution of body water between the intracellular (ICW) and extracellular (ECW) compartments. This parameter is of particular importance in assessing early age-related muscle changes.

Hydration status has a direct impact on muscle function, performing many important physiological roles. Water is the main component of skeletal muscle, accounting for over 75% of its mass. Total body water (TBW) consists of two main components: intracellular water (ICW), representing the fluid within the body’s cellular mass and providing the environment for key metabolic processes and cellular homeostasis, whereas extracellular water (ECW) refers to the sum of interstitial fluid and plasma, which is essential for the transport of nutrients [1,2,3].

An imbalance between ICW and ECW is associated with a reduction in cell volume, which accompanies the ageing process and muscle weakness. This trend becomes particularly pronounced after the age of 70 [4].

The available literature indicates that this issue is both scientifically valid and highly significant. There is growing recognition of the importance of BIA data for describing variations in body composition according to age and sex, as well as the need for more effective use of large BIA datasets in population analyses. As a non-invasive method, BIA is particularly suitable for screening purposes [5,6].

The available evidence highlights the biological and clinical significance of body water components (TBW, ECW, ICW) and their relationship with muscle mass and function [7,8]. From a biological perspective, this is significant because muscle ageing is associated not only with a reduction in muscle mass but also with qualitative changes in muscle tissue, including disturbances in intracellular and extracellular water, chronic low-grade inflammation, extracellular matrix remodelling, fibrosis, and fat infiltration.

According to the latest definition proposed by the European Working Group on Sarcopenia in Older People (EWGSOP2, 2018), sarcopenia is characterised as a progressive and generalised disorder of skeletal muscle leading to adverse clinical outcomes, such as falls, fractures, and disability, with the main emphasis placed on reduced muscle strength rather than solely on muscle mass [9,10,11].

Certain adverse changes in body water distribution may occur earlier than a measurable decline in skeletal muscle index (SMI). Therefore, hydration-related indices may serve as early markers of muscle ageing and changes associated with the development of sarcopenia.

In the broader context of ageing research, these indices may provide a valuable complement to molecular studies, including genetic and epigenetic research, as they reflect manifestations of ageing at the tissue and body composition levels, where cellular processes become observable as measurable structural and functional changes.

This approach may also be relevant in various pathological conditions characterised by catabolism and tissue remodelling, e.g., in oncological diseases, although this requires further clinical analysis [12].

In studies on ageing and sarcopenia, particular attention has been paid to the ECW/TBW ratio as a potential marker of muscle quality, with evidence suggesting that adverse changes in this parameter may precede a decline in the SMI index [3]. Recent studies indicate that the interpretation of hydration indices is strongly dependent on the measurement method used and the characteristics of the study population, which justifies the need to conduct analyses on homogeneous cohorts using standard measurement protocols [2,13,14,15].

From a clinical perspective, the results of our study may support the earlier identification of adverse qualitative changes in skeletal muscle in ageing individuals, prior to a significant decline in the SMI index. This may be relevant for the prevention of sarcopenia, the selection of individuals for further diagnostic assessment, and the monitoring of intervention outcomes, including nutritional, physical, and therapeutic strategies.

At the same time, body water content indices obtained using BIA should be regarded as supplementary tools rather than standalone diagnostic criteria in the assessment of sarcopenia [16,17].

## 2. Aim of the Study

The aim of this study is to assess the variability of body water distribution indices (ECW/ICW, ECW/TBW) in relation to age and sex, and to evaluate their association with skeletal muscle mass index (SMI) after adjusting for body mass index (BMI). Furthermore, the study aims to determine their usefulness as markers of age-related qualitative changes in skeletal muscle. Two research questions have been formulated. Firstly, do the ECW/ICW and ECW/TBW ratios differ according to age and sex? Secondly, are these ratios associated with SMI when BMI is simultaneously taken into account in the analysis?

## 3. Study Population and Methods

The study was conducted between October and December 2025 at the Central Laboratory of the University of Health and Sports Sciences in Wrocław. A total of 188 participants took part in the study, all of whom gave their written informed consent to participate in the study.

All procedures performed in studies involving human subjects complied with the ethical standards of the institutional and/or national research committee, as well as with the 1964 Declaration of Helsinki and its subsequent amendments or comparable ethical standards.

The project received a favourable opinion from the Senate Committee on Research Ethics at the Wrocław University of Health and Sport Sciences No. 19/2025 (date of approval: 21 May 2025).

The older cohort comprised 71 women (mean age 71.61 ± 6.38 years) and 37 men (mean age 72.70 ± 4.70 years), all of whom were independent residents of Wrocław. The younger group comprised 40 female students (mean age 22.48 ± 0.93 years) and 40 male students (mean age 22.77 ± 1.79 years) from the same university. All study participants engaged in moderate or high-intensity physical activity at least twice a week as part of their university studies (younger participants) and the University of the Third Age (older participants). The active lifestyle of the study participants was also confirmed by skeletal muscle mass index (SMI) values, which averaged 8.4 kg/m^2^ in the group of male seniors and 7.0 kg/m^2^ in the group of female seniors. SMI values indicating reduced muscle mass were observed in only 3 participants.

Body composition analysis was performed using bioelectrical impedance analysis (BIA) with a Tanita MC-780 analyser (Tanita Corporation, Tokio, Japan). The analysis included the following variables: age, sex, and body composition parameters obtained via BIA, including body mass index (BMI), skeletal muscle mass index (SMI), total body water (TBW), extracellular water (ECW), and intracellular water (ICW). The BIA method is based on the measurement of tissue resistance and reactance, which allows these parameters to be estimated. Hydration parameters were recorded in both absolute (kg) and relative (%) values. Before the measurement, participants were required to remove all metal objects and electronic devices. Measurements were taken in the morning, on an empty stomach, and participants were instructed to avoid strenuous physical activity for 12–24 h prior to the measurement [5]. All participants provided a urine sample prior to the tests, kept their fluid intake to a minimum, avoided caffeine and alcohol, and did not take any diuretics.

The Tanita MC-780 body composition analyser is a standard device that uses multi-frequency measurement technology (three electrical frequencies), ensuring compliance with the EWGSOP2 guidelines (2019). The skeletal muscle mass index (SMI) is a key parameter in the diagnosis of sarcopenia. The most commonly used formula, in accordance with EWGSOP2 recommendations, is SMI = skeletal muscle mass of the limbs (kg)/height^2^ (m^2^). This index enables the assessment of the risk of sarcopenia [10]. According to EWGSOP2 criteria, low muscle mass is defined as SMI < 7.0 kg/m^2^ in men and <5.5 kg/m^2^ in women, with lower values indicating an increased risk of sarcopenia [9].

Two indicators of body water distribution were calculated from the raw data: ECW/ICW and ECW/TBW. These indicators serve as the main reference points for interpreting the study results, as they are increasingly recognised in the literature as indirect indicators of muscle quality and phenotypic changes associated with ageing. Since ICW reflects muscle cell mass, whilst ECW represents the sum of interstitial fluid and plasma in the extracellular compartment, the increase in the ECW/ICW ratio observed in older adults indicates a relative increase in non-contractile tissue, thus serving as an alternative measure of muscle quality.

The ECW/TBW ratio has also recently been proposed as an indicator of muscle quality; however, its clinical and biological interpretation requires further investigation [3,18].

### Statistical Methods

The study was analysed as a cross-sectional comparative study. Four study groups were defined according to age cohort and sex: older women, older men, female students, and male students. Sex was coded as a binary variable, where 0 denoted women and 1 denoted men. For the purposes of the two-factor linear models, age cohort was coded as 0 for the younger/student cohort and 1 for the older cohort. The main variables were skeletal muscle index (SMI), body mass index (BMI), age, sex, and two indicators of body water distribution obtained using the BIA method: ECW/ICW and ECW/TBW.

As ECW/TBW is commonly used as a relative indicator of the extracellular water fraction in clinical BIA studies, it was selected as the primary indicator of body water distribution. ECW/ICW was retained as a secondary indicator and was analysed in sensitivity models. This choice was also supported by the deterministic relationship between these two indices. Total body water is the sum of extracellular and intracellular water (TBW = ECW + ICW). Therefore, if x denotes the ECW/ICW ratio, the ECW/TBW ratio can be expressed as

cECW/TBW=ECW/(ECW+ICW)=(ECW/ICW)/(1+ECW/ICW)=x/(1+x).

Conversely, ECW/ICW=(ECW/TBW)/(1−ECW/TBW).

This algebraic relationship explains why the two indices were expected to be highly correlated. Consequently, they were not treated as independent explanatory variables in the same main regression model.

Continuous variables were summarised as mean ± standard deviation (SD), and additionally as median with interquartile range (IQR), as several variables did not meet the assumption of normality. The sample mean and standard deviation were calculated as follows:

$$\bar{x}\;=\;\left(\frac{1}{n}\right)\;\sum_{i=1}^{n}x_{i}$$

(1) $$s=\sqrt{\frac{1}{n-1}\sum_{i=1}^{n}{\left(x_{i}-\bar{x}\right)}^{2}.}$$

The normality of the distributions of key variables was assessed using the Shapiro–Wilk test. As the assumption of normality was not consistently met, differences between the four study groups were primarily assessed using the Kruskal–Wallis test. In cases where the overall Kruskal–Wallis test was statistically significant, post hoc pairwise comparisons were performed using Dunn’s rank-sum test with Holm’s correction for multiple comparisons.

Given the characteristics of the data distribution and the presence of two clearly distinct age cohorts, Pearson’s correlation coefficients were not used for the primary analyses. Relationships between the variables were assessed using Kendall’s tau rank correlation coefficient.

Kendall’s tau was selected as a non-parametric measure of monotonic association, which does not require normally distributed variables and is more appropriate than Pearson’s correlation for the present data structure. Asymptotic z-statistics and two-sided *p*-values were used for inference.

Multivariate relationships with SMI were examined using ordinary least squares (OLS) regression. The OLS method was retained for the multivariate part of the analysis, as the research question required the inclusion of BMI, age, and sex. However, the model’s diagnostics were carefully assessed, and heteroscedasticity-robust HC3 standard errors were used for statistical inference in the main regression tables. The main model was defined as follows:

$${SMI}_{i}\;=\beta_{0}+\beta_{1}{\left(ECW/TBW\right)}_{i}+\beta_{2}{Sex}_{i}+\beta_{3}{Age}_{i}+\beta_{4}{BMI}_{i}+\varepsilon_{i}$$

In the sensitivity model, ECW/TBW was replaced by ECW/ICW:

$${SMI}_{i}\;=\beta_{0}+\beta_{1}{\left(ECW/ICW\right)}_{i}+\beta_{2}{Sex}_{i}+\beta_{3}{Age}_{i}+\beta_{4}{BMI}_{i}+\varepsilon_{i}$$

The model simultaneously accounting for both ECW/ICW and ECW/TBW was estimated solely as a diagnostic model for multicollinearity and was not used for the primary biological interpretation. Multicollinearity was assessed using the variance inflation factor (VIF), calculated as

$${\mathrm{VIF}}_{j}=1\;/\;(1-{R_{j}}^{2})$$

where ${R_{j}}^{2}$ is the coefficient of determination obtained after regressing the predictor j against the other predictors. VIF values above 10 were considered to indicate severe multicollinearity.

Regression diagnostics included an assessment of the normality of the residuals using the Shapiro–Wilk test, heteroscedasticity using the Breusch–Pagan and White tests, multicollinearity using VIF values, and outliers using Cook’s distance. Observations with a Cook’s distance greater than 4/N were flagged for review, but the main interpretation was based on the full dataset; the models were not destabilised by any single observation.

No formal a priori sample size calculation was available prior to data collection; therefore, the inferential analyses should be interpreted as exploratory. To characterise the detectable effect size for the available sample, a power sensitivity analysis was conducted. With *N* = 188, a tailed significance level of α = 0.05, and 80% statistical power, the study was able to detect correlation-type effects of approximately r = 0.20 or greater. For comparisons of four groups, the available sample size provided 80% power to detect a pooled effect of approximately Cohen’s f = 0.24, corresponding to η^2^ ≈ 0.056. In the context of regression analysis, the available sample size was sufficient to detect small incremental effects of approximately f^2^ = 0.04 for a single predictor examined in the presence of covariates. The absence of formal a priori calculations was taken into account when interpreting statistically non-significant results.

Physical activity was self-reported by the patients and was not available as a verified quantitative covariate; consequently, it was not included in the regression models. Similarly, food intake, 24 h fluid intake, medication use, comorbidities, oedema status, and menstrual or menopausal status were not available for statistical adjustment. Low skeletal muscle index (SMI) was defined according to the EWGSOP2 cut-off values of <7.0 kg/m^2^ for men and <5.5 kg/m^2^ for women. However, a full EWGSOP2 classification of probable, confirmed, or severe sarcopenia was not possible, as measurements of muscle strength and physical fitness were not collected.

All statistical tests were two-sided, and a *p*-value < 0.05 was considered statistically significant. Statistical analyses were performed using the Gretl software (version 2026a).

## 4. Results

The analysis included 188 participants: 71 older women, 37 older men, 40 female students, and 40 male students. The following results describe cross-sectional differences between age and sex groups and should not be interpreted as evidence of age-related changes in a longitudinal context.

### 4.1. Characteristics of the Study Sample and Non-Parametric Group Comparisons

Descriptive statistics by study group are presented in Table 1. Shapiro–Wilk tests revealed significant deviations from a normal distribution for SMI (W = 0.948, *p* < 0.001), BMI (W = 0.982, *p* = 0.015), age (W = 0.765, *p* < 0.001), ECW/ICW (W = 0.977, *p* = 0.004), and ECW/TBW (W = 0.975, *p* = 0.002). Consequently, overall comparisons between the four study groups were based on the Kruskal–Wallis test.

The Kruskal–Wallis tests revealed significant differences between the four groups in terms of BMI, SMI, ECW/ICW, and ECW/TBW (all *p* < 0.001). The same was observed for age, which was expected, as the study design deliberately compared a younger group (students) with an older group.

Post hoc comparisons confirmed that both ECW/ICW and ECW/TBW were higher in the older groups than in the corresponding student groups (Table 2). Older women had higher ECW/ICW and ECW/TBW values than female students, and older men had higher ECW/ICW and ECW/TBW values than male students (all *p*-values adjusted using the Holm method < 0.001). Women also exhibited higher values for both body water distribution indices than men in the same age group.

### 4.2. Two-Factor Linear Models for Age Cohort and Sex

Linear models with robust standard errors (HC3) were fitted, using age cohort, sex, and the age cohort × sex interaction as predictors (Table 3). These models were used to assess whether the group pattern suggested an interaction between age cohort and sex.

For ECW/ICW and ECW/TBW, the effects of age group and sex were statistically significant, whereas the interaction between age group and sex was not statistically significant. Thus, the difference between age groups in body water distribution indices was present in both women and men. For SMI, the interaction between age group and sex was statistically significant, indicating that the contrast between age groups in SMI differed by sex.

### 4.3. Kendall’s Rank Correlation Analysis

Kendall’s tau correlations are presented in Table 4. The analysis showed that the SMI index was negatively correlated with both ECW/ICW and ECW/TBW. The SMI index was positively associated with BMI and sex, reflecting higher SMI values in men, but was not significantly associated with age across the entire sample. Age was positively associated with both ECW/ICW and ECW/TBW.

The correlation between ECW/ICW and ECW/TBW was almost perfect (tau = 1.000, *p* < 0.001), indicating that both indices contain almost identical ranking information. This is consistent with their mathematical relationship and supports treating ECW/TBW as the primary indicator and ECW/ICW as a sensitivity indicator, rather than interpreting them as independent predictors in a simultaneous model.

### 4.4. Multicollinearity Diagnostics

The SMI model incorporating both ECW/ICW and ECW/TBW was estimated solely for the purpose of diagnosing multicollinearity (Table 5). The VIF values for both hydration indices exceeded 200, confirming severe multicollinearity and explaining the unstable, opposite signs of the regression coefficients observed in the simultaneous model. Consequently, this model was not used for the main conclusions.

### 4.5. Regression Models for SMI

As ECW/ICW and ECW/TBW were almost collinear, ECW/TBW was used as a representative indicator of body water distribution in the basic regression analysis, whilst ECW/ICW was used in a separate sensitivity model. The results are presented in Table 6. Inference was based on heteroscedasticity-robust HC3 standard errors.

In the baseline model, the ECW/TBW ratio was negatively associated with SMI after adjusting for BMI, age, and sex (*β* = −15.630, robust 95% CI: −21.415 to −9.846, *p* < 0.001). BMI and male sex were positively associated with SMI, whilst age was not statistically significant after adjustment. A sensitivity analysis using the ECW/ICW ratio yielded the same significant finding: the ECW/ICW ratio was negatively associated with the SMI index after adjusting for BMI, age, and sex (*β* = −4.702, robust 95% CI: −6.500 to −2.903, *p* < 0.001). These results should be interpreted as adjusted cross-sectional associations, rather than as evidence of independent causal effects or early diagnosis of sarcopenia.

### 4.6. Regression Diagnostics

In none of the SMI models was the hypothesis of normality of the residuals rejected. The Breusch–Pagán and White tests indicated heteroscedasticity; therefore, robust HC3 standard errors were used for inference. Although several observations exceeded the conventional Cook’s distance threshold of 4/N, the maximum Cook’s distance remained below 1 in both models, and the overall direction and significance of the key relationships involving body water distribution indices remained stable (Table 7).

### 4.7. Low SMI According to EWGSOP2 Cut-Off Values

Low SMI was assessed using EWGSOP2 cut-off values of <7.0 kg/m^2^ for men and <5.5 kg/m^2^ for women. Full classification of sarcopenia according to EWGSOP2 was not possible, as muscle strength and physical performance were not assessed (Table 8).

### 4.8. Summary of Statistical Results

1. Non-parametric group comparisons showed significant differences between the four study groups in BMI, SMI, ECW/ICW, and ECW/TBW. Older participants had higher ECW/ICW and ECW/TBW values than the corresponding student groups. In both age cohorts, women exhibited higher values for both body water distribution indices than men.
2. Kendall’s rank correlation analysis confirmed that ECW/ICW and ECW/TBW were positively associated with age and BMI and negatively associated with SMI. SMI was not significantly associated with age in the full sample when assessed using Kendall’s tau, whereas sex and BMI showed stronger associations with SMI.
3. ECW/ICW and ECW/TBW contained almost identical rank information and produced severe multicollinearity when included together in a single SMI regression model. Therefore, ECW/TBW was used as the primary body water distribution index, whereas ECW/ICW was used as a sensitivity index. In separate regression models, both indices remained negatively associated with SMI after adjustment for BMI, age, and sex, with inference based on heteroscedasticity-robust HC3 standard errors.

## 5. Discussion

A particularly noteworthy finding of this study is that age was more clearly associated with body water distribution indices than with SMI. Kendall’s rank correlations showed positive associations between age and both ECW/ICW and ECW/TBW, whereas SMI was not significantly associated with age in the full sample. This may suggest that muscle ageing is more clearly reflected in BIA-derived body water distribution indices than in SMI alone.

The study by Hioka et al. also demonstrated that the ECW/TBW ratio increases with age in community-dwelling women and that the adverse effects of ageing may become apparent earlier for the ECW/TBW ratio than for the SMI. Our analysis extends these findings, demonstrating a similar pattern in a dataset comprising both women and men, as well as two distinctly different age cohorts assessed under a single, standardised measurement protocol [3].

In the study by Ohashi et al., an increase in the ECW/ICW ratio was observed with age, which the authors attributed primarily to a relative decrease in intracellular water volume, i.e., a decline in ICW. In our sample, older participants showed significantly higher ECW/ICW values compared to the student groups, which may indicate that this ratio changes significantly with age [4].

Hetherington-Rauth et al., on the other hand, demonstrated that hydration parameters measured using the BIA method are associated with muscle performance, with ICW being a better predictor of muscle function than ECW [7]. Nakamura et al. demonstrated that a higher ECW/ICW ratio is associated with reduced handgrip strength and poorer functional status in patients with heart failure. Although functional assessments were not included in the present study, our results fit well with this line of interpretation, as they indicate that an unfavourable hydration profile co-occurs with a less favourable muscle profile and is strongly associated with age [8].

Studies conducted by Choi and Zhang [2,13] also provide important methodological context. Choi demonstrated that the ECW/TBW ratio is sensitive to measurement conditions and can be compared between standing and supine positions with high agreement [13]. Zhang demonstrated that even short-term changes in hydration status affect ECW, ICW, ECW/TBW, and ECW/ICW, with these effects varying by sex [2]. This has two important implications for the interpretation of our results. Firstly, it confirms that hydration parameters are indeed sensitive indicators. Secondly, it justifies the importance of using a standard measurement protocol and a highly sensitive analyser. It appears that in analytical practice, it is important to assess not only the ICW and ECW values themselves, but also their ratio. These are regarded as indicators of: nutritional status, cell membrane integrity, water–electrolyte balance, or the presence of inflammatory or catabolic processes.

The available literature increasingly suggests that body water indices obtained using BIA can also be used indirectly to assess muscle quality and nutritional status. Silva et al. emphasise the need for large-scale, integrated BIAs that account for age- and sex-related variability [5]. Branco et al. describe the growing importance of BIA and hydration parameters, whilst Zhang et al. highlight the prognostic value of the ICW/TBW ratio in oncology [12,14].

Our results suggest that BIA may have particular utility in population-based studies of muscle mass and related parameters. It appears that the concept of muscle quality should extend beyond the mere assessment of muscle mass or strength, reflecting the structural and metabolic integrity of the tissue at the cellular level [16,17].

Many guidelines for the diagnosis of sarcopenia consistently emphasise that muscle strength, muscle mass, and physical performance are closely interrelated in this condition. Numerous age- and sarcopenia-related changes primarily affect skeletal muscle and include, among others, changes in fat distribution, ectopic fat accumulation, chronic low-grade inflammation, structural muscle remodelling, a decline in muscle mass and function, and mitochondrial dysfunction. These processes have a significant impact on muscle metabolism and insulin sensitivity. Numerous studies have shown that these changes are associated not only with ageing but also with reduced levels of physical activity. Physical exercise has the potential to strengthen and regulate multiple systems simultaneously by inducing the production and release of myokines, which play a key role in maintaining overall metabolic homeostasis [19,20].

An additional risk to the ageing body may be the co-occurrence of sarcopenia and osteoporosis, referred to as osteosarcopenia. Defining osteosarcopenia as a distinct syndrome is crucial, as it provides a comprehensive picture of the musculoskeletal system, taking into account the importance of muscle health. This integrated condition significantly increases the risk of falls, fractures, hospitalisation, and mortality among older patients with chronic diseases, thereby increasing the medical, economic, and social burden [19,20,21].

An innovative aspect of this study is the combination of two complementary perspectives. On the one hand, the dataset is population-based and practical, as it derives from routine measurements obtained using the Tanita MC-780 analyser. On the other hand, the analysis is sufficiently comprehensive to demonstrate not only mean differences between groups, but also the structure of correlations and the behaviour of multivariate models.

A second important innovative feature is the specific composition of the study sample, in which both younger and older individuals of both sexes were analysed within the same experimental framework. Many previous studies have focused either on a single population of older adults or on a single sex [1,22,23]. In contrast, the inclusion of both younger and older women and men in a single dataset allows for a more direct observation of the ageing pattern.

A third important finding is the identification of very strong multicollinearity between ECW/ICW and ECW/TBW. This suggests that both variables can be considered closely related, partially interchangeable indicators.

Another innovative aspect of the study is the combination of two age cohorts and both sexes within a single, homogeneous dataset, assessed using the same Tanita MC-780 analyser. This enables the simultaneous assessment of the effects of age and sex, as well as the relationship between hydration indices and the skeletal muscle index (SMI). Many previous studies have used different protocols, devices, and isolated populations [3,8].

Importantly, the present results suggest that body water distribution indices may be more sensitive markers of muscle ageing than muscle mass alone.

Furthermore, these results may have practical implications, suggesting that the assessment of body water distribution using the methodologically simple and non-invasive BIA technique may be applicable in population and screening studies.

It should be emphasised, however, that the results of tests using the BIA method are influenced by a wide range of confounding factors; therefore, it can only be used for screening and population-based studies.

This study involved generally healthy individuals who had high or moderate levels of physical activity, and the prevalence of obesity was relatively low. Future studies should extend these analyses to overweight and obese populations, as well as to individuals with low levels of physical activity. It would also be worth taking into account additional structural and functional factors that may contribute to the risk of sarcopenia or osteosarcopenia. It would certainly be worth considering conducting an analysis using other diagnostic methods for assessing body composition, such as DEXA.

### Limitations

Our study is a typical cross-sectional study, which largely precludes causal inferences; there is also a lack of measurements of muscle strength and indicators of physical fitness. The phase of the menstrual cycle was not taken into account in the study of young women, which may also affect hydration results, and no precise quantitative analysis of daily fluid intake was carried out. The level of physical activity was also not quantified, but assessed declaratively through the systematic participation of the study participants in sports activities. Another factor limiting the conclusions drawn was the use of the BIA method to analyse body composition.

## 6. Conclusions

1. The present study indicates that body water distribution indices derived from BIA, particularly ECW/TBW and ECW/ICW, are closely related to age- and sex-dependent differences in body composition. These indices appear to reflect age-related variation in skeletal muscle phenotype more clearly than SMI alone in this physically active sample.
2. The association between body water distribution indices and SMI persisted after adjustment for BMI, age, and sex, supporting their role as complementary indicators of skeletal muscle phenotype. However, ECW/ICW and ECW/TBW should not be interpreted as independent markers in the same model, because they contain nearly identical information.
3. Overall, the results support the usefulness of BIA-derived body water distribution indices for describing cross-sectional patterns relevant to skeletal muscle ageing.

## Figures and Tables

**Table 1:** Descriptive characteristics of the study participants and results of the Kruskal–Wallis tests for the four study groups.

Variable	Older Women (*n* = 71)	Older Men (*n* = 37)	Female Students (*n* = 40)	Male Students (*n* = 40)	H	*p*-Value
Age [years]	71.61 ± 6.38 71.00 [67.50; 76.00]	72.70 ± 4.70 73.00 [69.00; 76.00]	22.48 ± 0.93 23.00 [22.00; 23.00]	22.75 ± 1.78 23.00 [21.00; 24.00]	138.47	<0.001
BMI [kg/m^2^]	27.28 ± 3.74 26.90 [24.80; 29.25]	27.89 ± 4.08 27.80 [25.20; 31.40]	21.85 ± 3.25 21.25 [19.82; 23.00]	24.06 ± 2.90 23.90 [22.53; 25.62]	62.38	<0.001
SMI [kg/m^2^]	7.02 ± 0.63 6.92 [6.58; 7.41]	8.40 ± 1.00 8.38 [7.67; 9.10]	6.60 ± 0.59 6.47 [6.24; 6.91]	9.08 ± 0.75 9.14 [8.61; 9.62]	116.62	<0.001
ECW/ICW	0.885 ± 0.065 0.888 [0.851; 0.920]	0.800 ± 0.047 0.792 [0.765; 0.821]	0.697 ± 0.038 0.694 [0.664; 0.712]	0.636 ± 0.051 0.640 [0.615; 0.678]	149.50	<0.001
ECW/TBW	0.469 ± 0.019 0.470 [0.460; 0.479]	0.444 ± 0.014 0.442 [0.433; 0.451]	0.410 ± 0.013 0.410 [0.399; 0.416]	0.388 ± 0.020 0.390 [0.381; 0.404]	149.50	<0.001

Values are presented as mean ± SD and median [IQR]. H denotes the Kruskal–Wallis test statistic.

**Table 2:** Dunn’s post hoc pairwise comparisons for body water distribution indices with *p*-values adjusted using the Holm method.

Variable	Comparison	Dunn’s z Statistic	Holm-Corrected *p*-Value
ECW/ICW	Older women vs. older men	3.60	<0.001
ECW/ICW	Older women vs. female students	8.45	<0.001
ECW/ICW	Older women vs. male students	11.15	<0.001
ECW/ICW	Older men vs. female students	4.12	<0.001
ECW/ICW	Older men vs. male students	6.46	<0.001
ECW/ICW	Female students vs. male students	2.39	0.017
ECW/TBW	Older women vs. older men	3.60	<0.001
ECW/TBW	Older women vs. female students	8.45	<0.001
ECW/TBW	Older women vs. male students	11.15	<0.001
ECW/TBW	Older men vs. female students	4.12	<0.001
ECW/TBW	Older men vs. male students	6.46	<0.001
ECW/TBW	Female students vs. male students	2.39	0.017

Positive values indicate that the first group had higher ranks than the second group. Because ECW/TBW is a monotonic transformation of ECW/ICW, both indices produced identical rank-based post hoc patterns.

**Table 3:** Two-factor linear models for age cohort, sex, and their interaction.

Result	Older Age Group *β*	*p*-Value	Male Sex *β*	*p*-Value	Older Cohort × Male Sex *β*	*p*-Value
SMI [kg/m^2^]	0.423	<0.001	2.477	<0.001	−1.104	<0.001
BMI [kg/m^2^]	5.424	<0.001	2.205	0.002	−1.595	0.137
ECW/ICW	0.189	<0.001	−0.061	<0.001	−0.025	0.101
ECW/TBW	0.059	<0.001	−0.022	<0.001	−0.003	0.597

Reference categories were the younger/student cohort and female sex. Therefore, the intercept corresponds to female students, the older cohort coefficient represents the difference between older women and female students, the male sex coefficient represents the difference between male and female students, and the interaction term indicates whether the difference between older and younger participants differs by sex. Inference was based on heteroscedasticity-robust HC3 standard errors.

**Table 4:** Kendall’s tau rank correlations in the full sample (*N* = 188).

Variables	Kendall’s Tau Coefficient	*p*-Value
SMI vs. ECW/ICW	−0.196	<0.001
SMI vs. ECW/TBW	−0.196	<0.001
SMI and sex	0.620	<0.001
SMI and BMI	0.367	<0.001
SMI and age	0.001	0.980
ECW/ICW vs. ECW/TBW	1.000	<0.001
ECW/ICW vs. age	0.563	<0.001
ECW/ICW and BMI	0.350	<0.001
ECW/TBW and sex	−0.361	<0.001
ECW/TBW and age	0.563	<0.001
ECW/TBW and BMI	0.350	<0.001
Sex and age	−0.080	0.191
Sex and BMI	0.057	0.346
Age and BMI	0.397	<0.001

Sex was coded as 0 = female and 1 = male. Kendall’s tau coefficient was used as the primary measure of correlation; Pearson’s correlation was not used to draw conclusions.

**Table 5:** VIF values in the diagnostic model including both ECW/ICW and ECW/TBW.

Predicted	VIF
ECW/ICW	205.91
ECW/TBW	205.95
Sex	1.76
Age	4.17
BMI	1.65

Diagnostic model: SMI ~ ECW/ICW + ECW/TBW + sex + age + BMI. VIF values > 10 indicate severe multicollinearity.

**Table 6:** Separate OLS regression models for SMI using ECW/TBW and ECW/ICW.

Predicted Variable	*β*	Robust Standard Error	95% CI	*p*-Value	VIF
**Base model: ECW/TBW**					
Intercept	8.824	1.037	6.778 to 10.871	<0.001	
ECW/TBW	−15.630	2.932	from −21.415 to −9.846	<0.001	5.54
Sex (men vs. women)	1.232	0.108	1.019 to 1.444	<0.001	1.75
Age [years]	−0.002	0.003	from −0.009 to 0.005	0.529	4.17
BMI [kg/m^2^]	0.204	0.009	0.186 to 0.222	<0.001	1.59
**Sensitivity model: ECW/ICW**					
Intercept	5.720	0.484	4.765 to 6.675	<0.001	
ECW/ICW	−4.702	0.912	from −6.500 to −2.903	<0.001	5.54
Sex (men vs. women)	1.253	0.107	1.042 to 1.464	<0.001	1.76
Age [years]	−0.003	0.003	from −0.010 to 0.003	0.309	4.09
BMI [kg/m^2^]	0.205	0.009	from 0.186 to 0.223	<0.001	1.62

Basic model: SMI ~ ECW/TBW + sex + age + BMI; *R*^2^ = 0.911, adjusted *R*^2^ = 0.909, F(4,183) = 466.97, *p* < 0.001. Sensitivity model: SMI ~ ECW/ICW + sex + age + BMI; *R*^2^ = 0.906, adjusted *R*^2^ = 0.904, F(4,183) = 440.41, *p* < 0.001.

**Table 7:** Diagnostics for the primary and sensitivity SMI regression models.

Model	Shapiro–Wilk *p*	Breusch–Pagan *p*-Value	White’s *p*-Value	Maximum Cook’s D	Cook’s D > 4/N
Basic SMI model with ECW/TBW	0.381	0.030	<0.001	0.425	8
Sensitivity SMI model with ECW/ICW	0.630	0.041	<0.001	0.357	9

Cook’s D-statistic > 4/N indicates the number of observations selected for inspection using the conventional 4/N rule.

**Table 8:** Low SMI according to EWGSOP2 cut-off values by sex and age cohort.

Group	*N*	Low SMI, *n*	Low SMI, %
Older women	71	0	0.0%
Older men	37	3	8.1%
Female students	40	0	0.0%
Male students	40	0	0.0

Only three participants met the criterion for a low sex-specific SMI score, and all of them were elderly men. No elderly women, female students, or male students reached the threshold for a low SMI score. These results should not be interpreted as estimates of the prevalence of probable, confirmed or severe sarcopenia, as the functional components required by EWGSOP2 were not available.

## Data Availability

The data presented in this study are not publicly available due to ethical and privacy restrictions. The study involves human participants, and the informed consent obtained did not include permission for public data sharing to protect participant confidentiality in accordance with the General Data Protection Regulation (GDPR).
